# Accurate 3D Positioning for a Mobile Platform in Non-Line-of-Sight Scenarios Based on IMU/Magnetometer Sensor Fusion

**DOI:** 10.3390/s18010126

**Published:** 2018-01-04

**Authors:** Hendrik Hellmers, Zakaria Kasmi, Abdelmoumen Norrdine, Andreas Eichhorn

**Affiliations:** 1 Institut für Geodäsie, Technische Universität Darmstadt, FG Geodätische Messsysteme und Sensorik, Franziska-Braun-Straße 7, 64287 Darmstadt, Germany; hen.hellmers@gmx.de (H.H.); eichhorn@geod.tu-darmstadt.de (A.E.); 2 Department of Mathematics and Computer Science, Freie Universität Berlin, Takustraße 9, 14195 Berlin, Germany; 3 Institut für Baubetrieb, Technische Universität Darmstadt, El-Lissitzky-Straße 1, 64287 Darmstadt, Germany; a.norrdine@baubetrieb.tu-darmstadt.de

**Keywords:** mobile platform, robotic, indoor positioning, magnetic field, Kalman filter, magnetometer, pressure sensor, barometer, MILPS

## Abstract

In recent years, a variety of real-time applications benefit from services provided by localization systems due to the advent of sensing and communication technologies. Since the Global Navigation Satellite System (GNSS) enables localization only outside buildings, applications for indoor positioning and navigation use alternative technologies. Ultra Wide Band Signals (UWB), Wireless Local Area Network (WLAN), ultrasonic or infrared are common examples. However, these technologies suffer from fading and multipath effects caused by objects and materials in the building. In contrast, magnetic fields are able to pass through obstacles without significant propagation errors, i.e. in Non-Line of Sight Scenarios (NLoS). The aim of this work is to propose a novel indoor positioning system based on artificially generated magnetic fields in combination with Inertial Measurement Units (IMUs). In order to reach a better coverage, multiple coils are used as reference points. A basic algorithm for three-dimensional applications is demonstrated as well as evaluated in this article. The established system is then realized by a sensor fusion principle as well as a kinematic motion model on the basis of a Kalman filter. Furthermore, a pressure sensor is used in combination with an adaptive filtering method to reliably estimate the platform’s altitude.

## 1. Introduction

Localization and navigation in overbuild areas are very challenging due to multipath effects. However, in common approaches, low cost sensors such as smartphone’s inertial sensors enable observations of motion changes, distances or azimuths in indoor environments. Nevertheless, indoor navigation approaches are restricted by the inertial sensors’ drift errors as well as the inaccuracies of the compasses. In contrast to the pedestrian localization, the most systems for tracking objects in hospitals or autonomous robots in factory halls are developed in the context of wheeled vehicles and use infrastructure based positioning systems. Modern infrastructures are, however, based upon electromagnetic or ultrasonic waves and hence influenced by shading effects and signal delay. An enclosing technology for universal solutions does not ultimately exist. A magnetic signal based Indoor Local Positioning System (MILPS) has been introduced to overcome these effects . This system has been created and further developed in the scope of academic research.

Although magnetic fields do not suffer from shading or multipath effects and so enable an efficient localization within Non-Line of Sight (NLoS) scenarios, they show a limited coverage area, since the magnetic field strengths decrease rapidly with the distance. Therefore, inertial sensors are used to cover areas, which cannot be reached from the MILPS, by merging accelerations and angular rates with magnetic measurements. Furthermore, air pressure observations are utilized to deliver information about altitude changes.

This contribution focus on a reliable 3D indoor positioning algorithm for moving platforms utilizing high rated inertial data as well as artificially generated magnetic fields. The suggested method does not suffer from shading or multipath effects and so enable an efficient localization within NLoS scenarios. Furthermore, the suggested method can be deployed in harsh environments such as mines or bunkers with thick walls.

A detailed description of the underlying kinematic motion model as well as of the Kalman filtering as sensor fusion method is given. Furthermore, the complete prototype is tested and evaluated within a real-world environment.

The article is organized as follows: Section 2 shows a summary of related work, while the applied system overview follows in Section 3. Section 4 explains in detail the estimation of the position and orientation by fusing magnetic with inertial data. The practical implementation of the Inertial Measurement Unit (IMU)/magnetometer integration as well as the reached tracking results are given in Section 5. Finally, the article is concluded in Section 6.

## 2. Related Work

Tracking systems usually use an emitter/sensor pair to determine a position of an object as well as measurement techniques such as the Time-of-Flight (ToF) [1] and the Angle-of-Arrival (AoA) [2]. They enable to determine distances, azimuths and elevation angles based on absolute time measurements. In contrast, the Time-Difference-of-Arrival (TDoA) allows for calculating the difference in distances between the target and two reference stations by using the difference in arrival time [3].

Various indoor localization approaches as well as technologies are described in [4], while Ref. [5] applies the Ultra Wide Band (UWB) technique—characterized by high frequency electromagnetic impulses—initially for a precise positioning; meanwhile, Refs. [6,7] provide reliable solutions in the range of decimeters for object tracking in warehouses and factory halls. Furthermore, a combination of UWB with inertial measurements is presented in [8].

Infrared signals technologies for pedestrian localization as well as for mobile robots are introduced in [9,10]. By these approaches, hyperbolic cuts are used according to the measures of time differences to various reference antennas. In contrast to the electromagnetic case, an ultrasonic based approach is developed by [11]. This approach uses measurements of the propagation time to enable the computation of distances as well as ensuring a three-dimensional tracking, whereby an accuracy range of a few millimetres is reached. Another localization system using ultrasound by measuring distances between active and passive sensors is also presented in [12]. Image Recognition-Based Positioning (IRBP) is a promising approach for indoor localization. Dabove et al. demonstrated that Inertial Navigation System (INS) measurements can be coupled with image-based methods for smartphone localization [13].

Although the mentioned approaches enable a tracking within indoor environments, the underlying technologies are limited due to disturbances, multipath effects or bad lighting conditions. Consequently, magnetic field based systems were introduced by [5,14] to facilitate the realization of applications within NLoS scenarios. The development of MILPS is described in [15] and the capability of an IMU/magnetometer integration is manifested in [16]. In addition, experimental results concerning simple motions inside a building corridor are demonstrated in [17,18].

## 3. System Description

**Figure 1:**
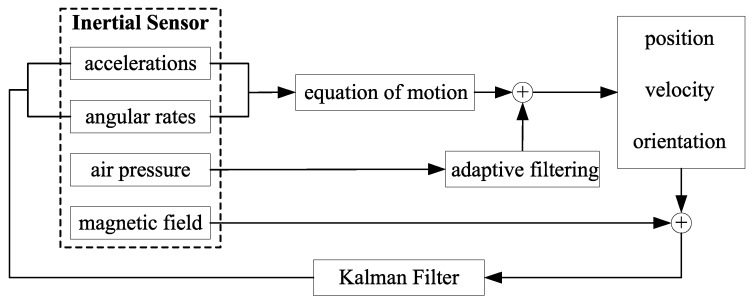
Flow diagram of the positioning algorithm.

This section gives an overview about the system architecture as well as the performed signal processing of the collected data based on the IMU/magnetometer sensor unit. Figure 1 shows the complete fusion algorithm within a flow diagram. Acceleration and angular rate measurements from a Microelectro-Mechanical Systems (MEMSs) inertial sensor enables the determination of the object position, velocity and orientation (see Section 3.3 and Section 4). In addition, measured air pressure delivers further information about the height. The magnetic field measurements from MILPS are applied to reduce position drifts (see Section 3.2). The applied sensor fusion enables to estimate the system’s state, which is composed of a three-dimensional position, velocity and orientation. Finally, a fusion of inertial and magnetic field data is realized by the method of Kalman filter (see Section 4). The magnetic data are generated by various coils.

### 3.1. Magnetic Signals

MILPS enables the localization of a user by using coils as reference points, whereby the coils are excited by a direct current that generates static magnetic fields whose intensities degrease with the distance to the coils [14]. In this context, the distance to a reference coil is equal to:

(1) $$d=\sqrt[{3}]{\frac{\mu_{0}\cdot I\cdot N\cdot A}{|B|.4\pi }\times \sqrt{1+3\cdot \sin^{2}\left(\phi \right)}},$$

whereby *d* defines the distance to the coil as a function of the magnetic field intensity |B|, *N* is the number of wire turns, *I* is the current running through the coil, *A* is the base area, and ϕ is the elevation angle. In addition, $\mu_{0}$ is the permeability of free space that equals $1.26\times 10^{-6}N$/$A^{2}$. The elevation angle ϕ can be calculated as follows:

(2) $$\phi =\arctan\left(-\frac{3}{4}\cdot \tan\left(\theta \right)\pm \sqrt{{\left(\frac{3}{4}\cdot \tan\left(\theta \right)\right)}^{2}+\frac{1}{2}}\right),$$

where the variable θ describes the angle between the local magnetic field vector B and the horizontal plane, which are defined as follows [5]:

(3) $$\begin{array}{cc} \theta & =arcsin(\frac{B_{z}}{\left|B\right|}) \end{array}$$

(4) $$\begin{array}{cc} B & ={\left(B_{x}^{local},B_{y}^{local}B_{z}^{local}\right)}^{T}. \end{array}$$

**Figure 2:**
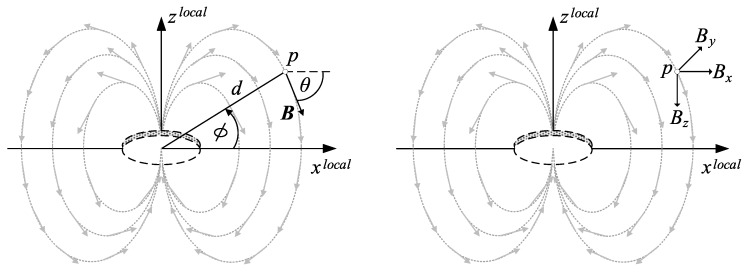
Magnetic field of a coil.

In this work, vectors are designated with bold and matrices are represented with upper case. Figure 2 illustrates the induced magnetic field at the point *p*. Besides the elevation angle Φ and the slope distance *d*, the point’s coordinates can be described by triaxial vector components [14]. Therefore, the local magnetic field B at point p(x,y,z) can be expressed by:

(5) $$B_{x}^{local}=\frac{3\cdot \mu_{0}\cdot I\cdot N\cdot A}{4\pi }\times \frac{\left(x-x_{i}\right)\cdot \left(z-z_{i}\right)}{{\sqrt{{\left(x-x_{i}\right)}^{2}+{\left(y-y_{i}\right)}^{2}+{\left(z-z_{i}\right)}^{2}}}^{5}}\;,$$

(6) $$B_{y}^{local}=\frac{3\cdot \mu_{0}\cdot I\cdot N\cdot A}{4\pi }\times \frac{\left(y-y_{i}\right)\cdot \left(z-z_{i}\right)}{{\sqrt{{\left(x-x_{i}\right)}^{2}+{\left(y-y_{i}\right)}^{2}+{\left(z-z_{i}\right)}^{2}}}^{5}}\;,$$

and

(7) $$B_{z}^{local}=\frac{\mu_{0}\cdot I\cdot N\cdot A}{4\pi }\times \frac{2\cdot {\left(z-z_{i}\right)}^{2}-{\left(x-x_{i}\right)}^{2}-{\left(y-y_{i}\right)}^{2}}{{\sqrt{{\left(x-x_{i}\right)}^{2}+{\left(y-y_{i}\right)}^{2}+{\left(z-z_{i}\right)}^{2}}}^{5}}\;,$$

where (x,y,z) and $\left(x_{i},y_{i},z_{i}\right)$ represent the unknown point’s as well as the $i^{th}$ coil’s coordinates.

The operating principle of MILPS, which includes creation, preprocessing and extraction of the necessary magnetic field information, is described in the following subsection.

### 3.2. MILPS—Magnetic Indoor Local Positioning System

MILPS generates artificial magnetic signals by using coils, which are driven by a pulsed Direct Current (DC). It enables a static 3D positioning in NLoS conditions with a signal range of about 12 m and a position accuracy of less than 0.5 m.

#### 3.2.1. Hardware Components

**Figure 3:**
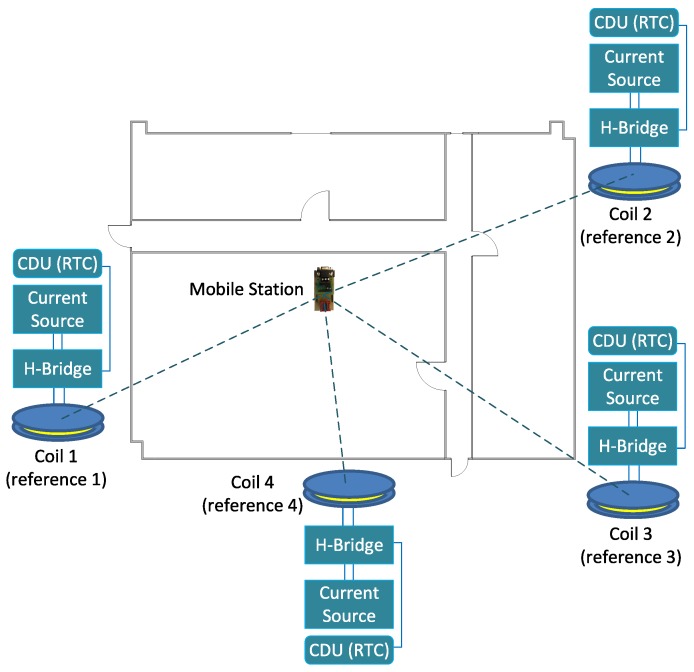
MILPS architecture.

MILPS is infrastructure-based and consists of coils with known positions (anchors) as well as a Mobile Station (MS). The MS includes a magnetometer to measure the magnetic field strength. The position of the MS is estimated by measuring the strengths of the magnetic fields generated by coils 1, 2 and 3. The Control Driver Unit (CDU) enables therefore the coil’s control via a H-bridge, in order to control the current direction (see Figure 3).

**Figure 4:**
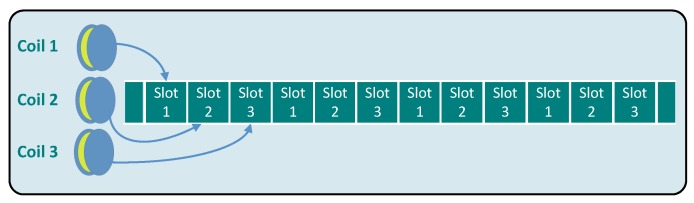
MILPS system synchronization scheme (TDMA). TDMA, time division multiple access.

MILPS follows a decentralized synchronization mechanism, which implements a periodic control of the coils and the MS using the Time Division Multiple Access (TDMA) scheme (see Figure 4). The MS is synchronized with the CDUs without the need of a synchronization bus or any kind of communication [15]. The control and the synchronization of the coils as well as of the MS is based on Real-Time Clocks (RTCs) and the Real-Time Operating System (RIOT-OS) [15].

This exemplary platform is employed in MILPS, whereas the mobile station and the reference points are equipped with real-time clocks (see Figure 3). This platform enables the generation of the DC-pulsed magnetic signals in predefined time slots with a period of time set to 0.3 s.

#### 3.2.2. Magnetic Signal Preprocessing and Position Calculation

The gathered raw magnetic field data should be preprocessed, since they are subjected to disturbances caused by the earth magnetic field or ferromagnetic materials and electrical appliances (e.g., fridge or elevator). Therefore, the influence of low frequency noise is eliminated by creating signals with respect to a known pattern. The overlying magnetic field conducted by the earth and other long periodic magnetic interferences are eliminated by switching the direction of the electrical current of each coil in polarity. The corresponding vector fields are characterized by equidistant direction changes and the same amount during the time interval $\Delta t_{coil}$.

**Figure 5:**
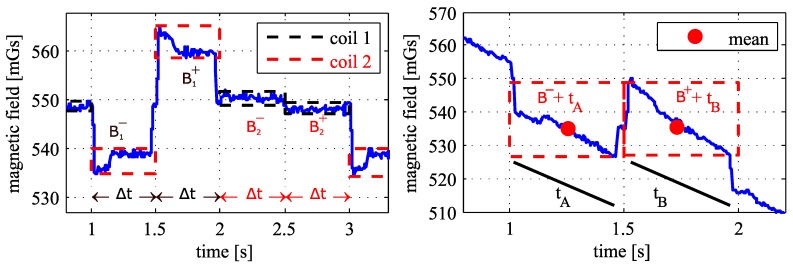
Magnetic field intensity at a static (**left**) and dynamic (**right**) magnetometer.

The left chart in Figure 5 shows an extract of the captured magnetic field intensity in the local *x*-direction from a static sensor with respect to two reference points. The signal polarity as well as intensity depends on the configuration between the object (sensor) and the corresponding coil. The local magnetic field components $B_{i}^{-}$ and $B_{i}^{+}$ describe the $i^{th}$ coil’s signal in positive and negative current directions, respectively. Assuming that low frequency interferences do not change during the switching interval Δt, a coil’s specific magnetic field $B_{i}$ can be calculated by taking the difference between the corresponding negative and positive signal sections. Therefore, the arithmetic means of two subsequent clusters (a cluster describes the measured signal during the time Δt) leads to elimination of high frequencies. The specific signal intensity of the $i^{th}$ coil is calculated as follows [5]:

(8) $$B_{i}=\frac{B_{i}^{+}-B_{i}^{-}}{2}.$$

The unknown position can be calculated by using Equations (1), (2) and (5)–(7). Although artificially generated magnetic fields enable an absolute positioning inside NLoS-scenarios, it necessitates the assumption of a constant signal at the surrounding fields, which means the object should be static during the measurement phase. However, varying disturbances are caused depending on the object’s velocity and direction, which consequently affects the measured signal at both subsequent observation clusters [19]. The right chart in Figure 5 shows an extract of a captured magnetic signal of a moving magnetic sensor (magnetometer). Biased clusters are caused by velocity, orientation changes, and kinematic effects, especially within 3D environments. However, these overlaying drifts can be assumed as linear approximations $t_{A}$ and $t_{B}$ by a constant velocity and moving direction during the switching time interval [18]. This effect can be eliminated by using the least squares method and the linear regression estimations per signal cluster in a first step [18].

Anchor-based localization systems are usually limited to a small area. However, an effective solution for large-scale indoor areas is generally not guaranteed. Therefore, reliable real-time applications require the utilization of IMUs for smooth localization.

### 3.3. Inertial Measurement Unit

The established localization system (MILPS) has a limited range; therefore, we use the IMU to overcome these limitations. We utilize the tactical grade IMU ADIS 16480 from *Analog Devices*, which is a 10-DoF sensor including a three-axial accelerometer, a gyroscope and a magnetometer, in order to ensure observations of the moving platform and MILPS. Additionally, a digital barometer delivers data of air pressure to get information about the altitude changes. Table 1 summarizes the relevant technical sensor features.

A specific real-time bias estimation is performed in addition to the sensor’s factory calibration, which ensures corrections of systematic effects. Therefore, initial constant biases of the accelerometer and the gyroscope readings are considered by computing the stationary offsets before each data acquisition. Thus, the usage of such IMUs enables the relative localization of a moving object by the integration of observed motion changes with respect to the sampling time. However, high frequency interferences caused by the sensor’s noise as well as deterministic platform vibrations must be eliminated by different steps of the digital signal processing, which are explained in the following sections. Furthermore, the extracted acceleration and angular rate measurements with respect to the IMU’s sensor axis should be transferred into a local reference frame.

#### 3.3.1. Inertial Signal Processing

In addition to the output noise of the inertial sensor, which can be described by statistical parameters, the observed inertial signals are influenced by deterministic overlays caused by high frequency platform vibrations. At the first step, a low-pass filtering of both the acceleration and angular rate measurements is performed by using the moving average algorithm. An adaptive peak detection method afterwards provides extractions of real motion changes. The moving average of the captured time series x is calculated by considering consecutive signal clusters. The output signal $\bar{x}$ at timestamp k is then given by [21]:

(9) $${\bar{x}}_{k}=\frac{1}{n}\cdot \sum_{i=k-j}^{k+j}x_{i}\;\;\;\mathrm{with}\;\;\;j=\left(n-1\right)/2\;\;\;\mathrm{and}\;\;\;n\in N,$$

and the corresponding variance is equal to:

(10) $$\sigma_{x_{k}}^{2}=\frac{\sum_{i=k-j}^{k+j}{\left(x_{i}-{\bar{x}}_{k}\right)}^{2}}{n-1}.$$

The cluster length *n* is set to 51 (n=51), which corresponds to a time interval of 0.25 s. The computed moving average applied to both acceleration and angular rate readings enables a high-frequency noise reduction as well as a precise angular rate at the timestamp *k*.

**Figure 6:**
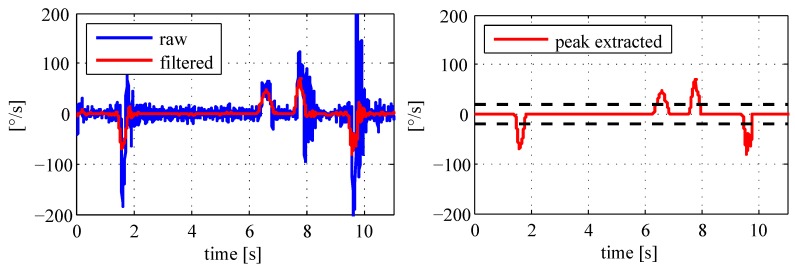
Processing of the angular rate data.

Figure 6 shows the proceeding of a filtering example using angular rate measures. A smoothed time series are observed, which are subject to an information extraction to obtain true motion changes. The peak detection in the signal time domain is applied utilizing predefined thresholds. The sample in Figure 6 is based on obvious direction changes during four explicit time intervals. Empirical values are thereby applied to extract all significant signal peaks, whereas other values are set to zero (see the right chart in Figure 6).

**Figure 7:**
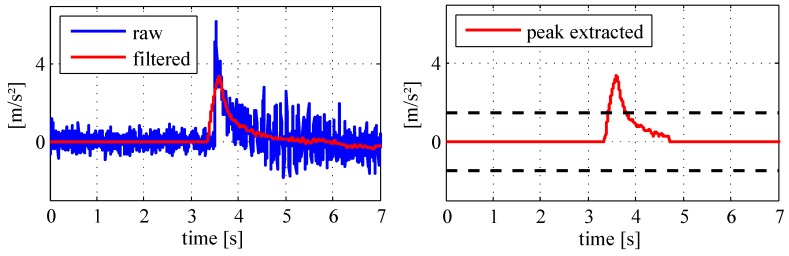
Processing of the acceleration data.

Figure 7 shows the equivalent information extraction of a specific acceleration signal. In this case, a velocity change of the moving object is observed between the time interval from 3.5 s to 4.5 s. Hence, the digital inertial data processing eliminates the static as well as dynamic noises caused by the platform vibrations, which finally leads to the specific angular rates $\left({\dot{\varphi }}_{x},{\dot{\varphi }}_{y},{\dot{\varphi }}_{z}\right)$ and the acceleration measurements $\left(\ddot{x},\ddot{y},\ddot{z}\right)$ at the timestamp *k* inclusive the corresponding variances. In the next subsection, an alternative adaptive filtering method is introduced to focus on variations in air pressure measurements.

#### 3.3.2. Adaptive Signal Filtering

In this work, high frequency components of inertial noise is reduced depending on the average order *n* by applying the moving average method explained in Section 3.3.1. This method describes a low-pass filter relaying on the convolution of the input signal x and the specific coefficients ω, whereas the output signal y at timestamp *k* is given by [22]:

(11) $$y_{k}=\omega {\left[k\right]}^{T}\cdot x\left[k\right],$$

whereby x[k] and ω are defined as follows:

(12) $$\begin{array}{cc} x\left[k\right] & ={\left(x_{k},x_{k-1},\ldots ,x_{k-n+1}\right)}^{T}, \end{array}$$

(13) $$\begin{array}{cc} \omega {\left[k\right]}^{T} & ={\left(\omega_{1},\omega_{2},\ldots ,\omega_{n}\right)}^{T}. \end{array}$$

In this case, pass- and stop ranges have to be defined a priori in order to eliminate the specified frequency components, so that constant characteristics within the frequency domain have to be assumed. The use of atmospheric measurements such as air pressure can be subject to rapid noise variations that require knowledge about the time variable filter characteristics. Therefore, the signal filtering in Equation (11) can be used to provide adaptive filter coefficients as well as enable the integration of pressure observations and positioning algorithms [23]. The practical implementation is based on a true-calculated comparison with regard to a reference signal at a known height. Thereby, the error signal $e_{k}$ is derived by computing the difference of the ideal signal d (desired signal) and the output signal $y_{k}$ at the time *k* as follows:

(14) $$e_{k}=d_{k}-y_{k}.$$

The function $e_{k}$, which describes the true-calculated difference with respect to the filter coefficients ω, can be expressed based on Equation (11) as follows:

(15) $$e_{k}\text{:}\;R^{n}\to R\;,\;e_{k}\left(\omega \left[k\right]\right)=d_{k}-\omega \left[k\right]\cdot x\left[k\right].$$

The optimal filter solution is then obtained by minimizing the squared errors of the signal based on the Least Mean Squares (LMS) algorithm:

(16) $$\min\;\left(e_{k}^{2}\right)\;\;\Rightarrow \;\;\frac{\partial e_{k}^{2}}{\partial \omega \left[k\right]}=0\;\;\mathrm{with}\;\;\omega \left[k\right]\neq 0.$$

The unbiased estimation is given by [23]:

(17) $$\omega \left[k+1\right]=\omega \left[k\right]+\nu \cdot e_{k}\cdot x\left[k\right]\;\;\mathrm{with}\;\;\nu >0,$$

where ν is an increment to approximate the solution.

**Figure 8:**
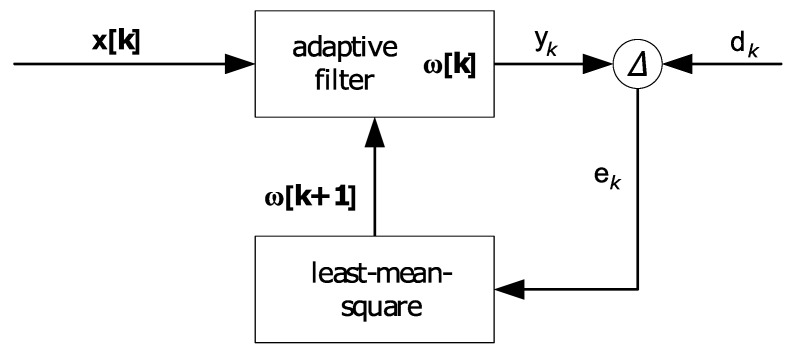
Flow diagram of the adaptive filter.

The method describes the LMS algorithm for an optimal adaptive filter estimation with respect to a reference station. Figure 8 shows the corresponding flow diagram. Hence, the unbiased estimation ω delivers an optimal filtering of the surrounding noise under the assumption that signal characteristics are equivalent at the reference as well as mobile station.

#### 3.3.3. Barometric Height Determination

The object’s altitude $z_{m}$ can be calculated with respect to the known reference high $z_{r}$ by using the barometric formula [24]:

(18) $$z_{m}=\frac{\ln\left(\frac{\rho_{m}}{\rho_{r}}\right)\cdot T\cdot R}{-m_{mol}\cdot g_{0}}+z_{r}.$$

In this context, $\rho_{m}$ and $\rho_{r}$ describe the air pressure at the mobile station and the reference height, respectively—*T* is the temperature in Kelvin, *R* is the universal gas constant, $m_{mol}$ is the mean atmosphere molar mass of the gas, and $g_{0}$ is the is the Earth gravitational constant. In our case, a constant pressure level of 1000 mbar is assumed at the reference station. For every timestamp *k*, the filter coefficients are determined according to Section 3.3.2 and applied to the pressure values ($\rho_{m}$) of the MS by applying Equation (18).

**Figure 9:**
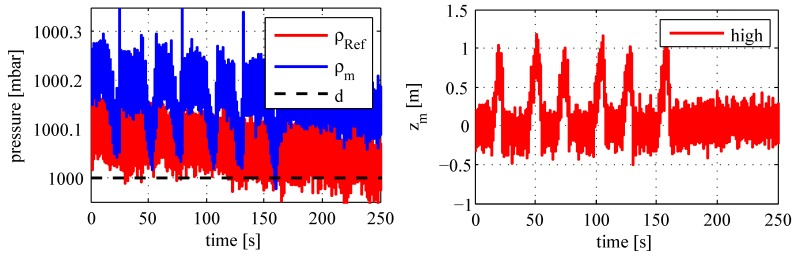
Determination of the barometric height.

Figure 9 shows captured pressure progressions (left chart) as well as the constant desired signal (right chart), which delivers the calculated z-coordinate as part of the system state. In contrast to the barometric high determination, the object’s entire state vector is computed by considering all processed inertial signals. However, this requires a transformation from the sensor’s coordinate system to the local frame with respect to the current orientation. Therefore, an overview about system transitions with sequential rotations is given in the next Section 4. This transformation is a relative state estimation as part of the positioning algorithm.

## 4. State Estimation

The three-dimensional kinematic state of the platform is described by the state transformation from timestamp *k* to timestamp k+1 using inertial data. This transformation delivers high-frequency solutions, which are the base of sensor fusion by using the absolute magnetic field observations in the Kalman filter process. The measured inertial raw signals as well as the magnetic field observations are thereby related to the IMU’s axes (in the following denoted as the body frame). Therefore, transformations into the absolute reference system (local frame) are required. The general principle of spatial rotations is described in Section 4.1, while the extension to a vector based representation is explained in Section 4.2. Section 4.3 covers the determination of relative state estimation, which builds the basis of the sensor integration method in Section 4.4.

### 4.1. Sequential Rotations

The spatial motion of the object with regard to the sensor’s orientation describes a $R^{3}$-rotation of the body frame in a local coordinate system for every discrete timestamp k. The orientation changes from state *k* to state k+1 are considered as relative rotations of the IMU’s reference axes and can be determined by the discrete angular rate time integration. In the following, the rotation matrix $R\in R^{3\times 3}$ characterizes the object’s current orientation. Depending on the angular rates ${\left({\dot{\varphi }}_{x},{\dot{\varphi }}_{y},{\dot{\varphi }}_{z}\right)}_{k+1}$, the orientation change during the sampling time interval is additionally expressed as $\Delta R\in R^{3\times 3}$.

A rotation in $R^{3}$ with respect to static coordinate axes is realized by the multiplication of a rotation matrix R with a vector $x\in R^{3\times 1}$. In the contrary, successive rotations are realized sequentially. Hence, the orientation change of the body frame $\Delta R_{k+1}\left({\dot{\varphi }}_{x},{\dot{\varphi }}_{y},{\dot{\varphi }}_{z}\right)$, which is referring to the present orientation $R_{k+1}$, leads to an orientation update with [25]:

(19) $$R_{k+1}=R_{k}\cdot \Delta R_{k+1}.$$

Applied to the current direction of travel $v_{k}\in R^{3\times 1}$, the direction update is given by utilizing the sequential rotations as follows [18]:

(20) $$v_{k+1}=R_{k}\cdot \Delta R_{k+1}\cdot R_{k}^{T}\cdot v_{k},$$

whereas $v_{k+1}$ describes the system’s direction change from state *k* to state k+1. In this case, the relation $R^{-1}=R^{T}$ for rotation matrices is implied. Basis of the established rotation matrices are the Euler angles, which separate the entire transformation space into three rotations per axis. To avoid nonlinearities, caused by the underlying trigonometric functions, a linear vector based method for $R^{3}$-rotations is introduced in the following Section 4.2.

### 4.2. Quaternions

In order to simplify the calculation of the rotation vectors in real-time applications, an angle representation with *quaternions* is used [26]. The usage of this special algebra enables avoiding nonlinearities and singularity problems, whereby a quaternion $q\in R^{4}$ is defined as:

(21) $$q={\left(q_{0},q_{1},q_{2},q_{3}\right)}^{T},$$

where $q_{0}$ describes the scalar and ${\left(q_{1},q_{2},q_{3}\right)}^{T}$ the vector part.

A general spatial rotation of a vector is defined as:

(22) $$p^{\prime }=q\cdot p\cdot q^{-1},$$

with $q\cdot q^{-1}={\left(1,0,0,0\right)}^{T}$. The vector part p is rotated into $p^{\prime }$. A multiplication of two quaternions is thereby given as follows:

(23) $$q\cdot p=\left(\begin{array}{c} q_{0}p_{0}-q_{1}p_{1}-q_{2}p_{2}-q_{3}p_{3}\\ q_{1}p_{0}+q_{0}p_{1}-q_{3}p_{2}+q_{2}p_{3}\\ q_{2}p_{0}+q_{3}p_{1}+q_{0}p_{2}-q_{1}p_{3}\\ q_{3}p_{0}-q_{2}p_{1}+q_{1}p_{2}+q_{0}p_{3} \end{array}\right).$$

Furthermore, two consecutive rotations—equivalent to Equation (19)—lead to the sequential quaternion multiplication:

(24) $$p^{\prime }=q^{I}q^{II}\cdot p\cdot {\left(q^{I}q^{II}\right)}^{-1},$$

where the second rotation $q^{II}$ refers to the axes, which are accordingly rotated by the first rotation $q^{I}$. With respect to Equation (23), the entire rotation can be finally expressed as a matrix-vector product:

(25) $$q^{I}\cdot q^{II}=\left(\begin{array}{cccc} q_{0}^{II} & -q_{1}^{II} & -q_{2}^{II} & -q_{3}^{II}\\ q_{1}^{II} & q_{0}^{II} & q_{3}^{II} & -q_{2}^{II}\\ q_{2}^{II} & -q_{3}^{II} & q_{0}^{II} & q_{1}^{II}\\ q_{3}^{II} & q_{2}^{II} & -q_{1}^{II} & q_{0}^{II} \end{array}\right)\cdot \left(\begin{array}{c} q_{0}^{I}\\ q_{1}^{I}\\ q_{2}^{I}\\ q_{3}^{I} \end{array}\right).$$

A quaternion-translation of the corresponding rotation matrix is given with [27]:

(26) $$R(q)=\left(\begin{array}{ccc} q_{0}^{2}+q_{1}^{2}-q_{2}^{2}-q_{3}^{2} & 2(q_{1}q_{2}-q_{0}q_{3}) & 2(q_{1}q_{3}+q_{0}q_{2})\\ 2(q_{2}q_{1}+q_{0}q_{3}) & q_{0}^{2}-q_{1}^{2}+q_{2}^{2}-q_{3}^{2} & 2(q_{2}q_{3}-q_{0}q_{1})\\ 2(q_{3}q_{1}-q_{0}q_{2}) & 2(q_{3}q_{2}+q_{0}q_{1}) & q_{0}^{2}-q_{1}^{2}-q_{2}^{2}+q_{3}^{2} \end{array}\right)\cdot \frac{1}{{\left|q\right|}^{2}}.$$

With respect to these relationships of reference system rotations, the complete description of the platform motion is shown in the next Section 4.3.

### 4.3. Equation of Motion

The object’s relative kinematic state includes the position (x,y,z), the velocity $\left(\dot{x},\dot{y},\dot{z}\right)$ and the orientation $\left(q_{0},q_{1},q_{2},q_{3}\right)$. This kinematic state is computed by utilizing the discrete integration of the current acceleration measurements $u=(\ddot{x},\ddot{y},\ddot{z})$ and angular rates $\dot{\varphi }=\left({\dot{\varphi }}_{x},{\dot{\varphi }}_{y},{\dot{\varphi }}_{z}\right)$ with respect to the sampling time. The complete system’s state vector is denoted as:

(27) $$x={\left(x,y,z,\dot{x},\dot{y},\dot{z},q_{0},q_{1},q_{2},q_{3}\right)}^{T}.$$

Since the system’s state is determined at every discrete timestamp *k*, a state transformation from state *k* to k+1 is carried out by applying the following motion equation [17]:

(28) $$x_{k+1}=\Phi \left({\dot{\varphi }}_{k+1}\right)\cdot x_{k}+C\cdot R\left(q_{k}\right)\cdot \left(u_{k+1}-g_{0}\right)\;\;\;\mathrm{with}\;\;\;\dot{\varphi },u\in R^{3},x\in R^{10},$$

whereby Φ stands for the transition matrix, C for the control matrix, u for the acceleration vector and $g_{0}$ for the earth’s gravity. The rotation matrix R is described as a function of the orientation quaternion q to rotate the triaxial acceleration of the body frame into a local frame. Furthermore, the transition matrix consists of four sub-matrices as follows:

(29) $$\Phi =\left(\begin{array}{cc} \Phi_{1,1} & \Phi_{1,2}\\ \Phi_{2,1} & \Phi_{2,2} \end{array}\right).$$

Matrix $\Phi_{1,1}$ represents a position and velocity transformation from state *k* to k+1 by utilizing the measured angular rates $\dot{\varphi }$ as small rotation changes relating to the current orientation. Assuming a high-frequency sample rate, the orientation changing matrix can be computed as follows [27]:

(30) $$\Delta R\left({\dot{\varphi }}_{k+1}\right)=\left(\begin{array}{ccc} 1 & -{\dot{\varphi }}_{z}\cdot \Delta t & {\dot{\varphi }}_{y}\cdot \Delta t\\ {\dot{\varphi }}_{z}\cdot \Delta t & 1 & -{\dot{\varphi }}_{x}\cdot \Delta t\\ -{\dot{\varphi }}_{y}\cdot \Delta t & {\dot{\varphi }}_{x}\cdot \Delta t & 1 \end{array}\right),$$

whereby the following approximation is used with respect to the sampling time Δt:

(31) sin(φ·Δt)≈φ·Δtandcos(φ·Δt)≈1.

The complete update of the velocity direction derived from Equation (20) is therefore equal to:

(32) $$V=R\left(q_{k}\right)\cdot \Delta R\left({\dot{\varphi }}_{k+1}\right)\cdot R^{T}\left(q_{k}\right),$$

which implies that the first transition sub-matrix can be given by the follow sub-matrix:

(33) $$\Phi_{1,1}=\left(\begin{array}{cccccc} 1 & 0 & 0 & \Delta t & 0 & 0\\ 0 & 1 & 0 & 0 & \Delta t & 0\\ 0 & 0 & 1 & 0 & 0 & \Delta t\\ 0 & 0 & 0 & V_{1,1} & V_{1,2} & V_{1,3}\\ 0 & 0 & 0 & V_{2,1} & V_{2,2} & V_{2,3}\\ 0 & 0 & 0 & V_{3,1} & V_{3,2} & V_{3,3} \end{array}\right).$$

Furthermore, the orientation change quaternion can be expressed as follows [27]:

(34) $$\Delta q=\left(\begin{array}{c} 1\\ \Delta t\cdot {\dot{\varphi }}_{x}/2\\ \Delta t\cdot {\dot{\varphi }}_{y}/2\\ \Delta t\cdot {\dot{\varphi }}_{z}/2 \end{array}\right).$$

Inserting Equation (34) in Equation (25) yields to the orientation’s update matrix $\Phi_{2,2}$:

(35) $$\Phi_{2,2}=\frac{\Delta t}{2}\cdot \left(\begin{array}{cccc} 2/\Delta t & -{\dot{\varphi }}_{x} & -{\dot{\varphi }}_{y} & -{\dot{\varphi }}_{z}\\ {\dot{\varphi }}_{x} & 2/\Delta t & {\dot{\varphi }}_{z} & -{\dot{\varphi }}_{y}\\ {\dot{\varphi }}_{y} & -{\dot{\varphi }}_{z} & 2/\Delta t & {\dot{\varphi }}_{x}\\ {\dot{\varphi }}_{z} & {\dot{\varphi }}_{y} & -{\dot{\varphi }}_{x} & 2/\Delta t \end{array}\right).$$

This equation enables the transformation of the quaternion $q_{k}$ to $q_{k+1}$. Finally, the control matrix C, which realizes the acceleration vector’s integration, is defined as follows:

(36) $$C=\left(\begin{array}{ccc} 1/2\cdot \Delta t^{2} & 0 & 0\\ 0 & 1/2\cdot \Delta t^{2} & 0\\ 0 & 0 & 1/2\cdot \Delta t^{2}\\ \Delta t & 0 & 0\\ 0 & \Delta t & 0\\ 0 & 0 & \Delta t\\ 0 & 0 & 0\\ 0 & 0 & 0\\ 0 & 0 & 0\\ 0 & 0 & 0 \end{array}\right).$$

Besides the estimation of the system current state $x_{k}$, the equation of motion delivers additional statements about the state vector’s accuracy. Therefore, the state’s corresponding Variance–Covariance Matrix (VCM) is computed by applying the variance propagation as follows:

(37) $${\left(\Sigma_{\mathrm{xx}}\right)}_{k+1}=\Phi \left({\dot{\varphi }}_{k+1}\right)\cdot {\left(\Sigma_{\mathrm{xx}}\right)}_{k}\cdot \Phi {\left({\dot{\varphi }}_{k+1}\right)}^{T}+C\cdot R\left(q_{k}\right)\cdot \Sigma_{\mathrm{uu}}\cdot {\left(C\cdot R\left(q_{k}\right)\right)}^{T}+A\cdot \Sigma_{\dot{\varphi }\dot{\varphi }}\cdot A^{T},$$

where $\Sigma_{\mathrm{xx}}$ is the state vector’s VCM. The matrices $\Sigma_{\mathrm{uu}}$ and $\Sigma_{\dot{\varphi }\dot{\varphi }}$ represent the accuracies of the acceleration and the angular rate measurements, respectively:

(38) $$\Sigma_{\mathrm{uu}}=\left(\begin{array}{ccc} \sigma_{\ddot{x}}^{2} & 0 & 0\\ 0 & \sigma_{\ddot{y}}^{2} & 0\\ 0 & 0 & \sigma_{\ddot{z}}^{2} \end{array}\right),$$

(39) $$\Sigma_{\dot{\varphi }\dot{\varphi }}=\left(\begin{array}{ccc} \sigma_{{\dot{\varphi }}_{x}}^{2} & 0 & 0\\ 0 & \sigma_{{\dot{\varphi }}_{y}}^{2} & 0\\ 0 & 0 & \sigma_{{\dot{\varphi }}_{z}}^{2} \end{array}\right).$$

The juncture matrix A ($A=\frac{\partial x}{\partial \dot{\varphi }}$) includes the partial derivations of Equation (28) with respect to $\dot{\varphi }$. In the following, the computed state as well as the corresponding VCM will be designated as predictions and denoted with a superscript minus.

### 4.4. Iterated Kalman Filter

The MILPS is deployed to compensate the instabilities of the dead reckoning system, whereby an IMU/magnetometer sensor fusion is used to support the system state $x^{-}$ as well as its variance-covariance matrix $\Sigma_{\mathrm{xx}}^{-}$. The method of Kalman filter is applied to update the predictions, if observations of the MILPS are available. An iterative executed modification is thereby carried out for reaching unbiased state estimations due to the arising of nonlinearities while using the observation equations [28]. Regarding Equations (5)–(7), the Iterated Kalman Filter (IKF) consists of the following observation model:

(40) $$f={\left(B_{x}\left(x,y,z\right)\;,\;B_{y}\left(x,y,z\right)\;,\;B_{z}\left(x,y,z\right)\right)}^{T},$$

which transforms the current position into the observation space with respect to the $i^{th}$ coil’s magnetic field. Depending on the current orientation, a transformation of the observed magnetic vector into the local coordinate frame must be performed as follows:

(41) $${\left(B_{x}^{local},B_{y}^{local},B_{z}^{local}\right)}^{T}=R\left(q_{k}\right)\cdot {\left(B_{x}^{body},B_{y}^{body},B_{z}^{body}\right)}^{T}.$$

The model linearization is realized with the *Jacobian matrix* H, which follows after *j* iterations at the state vector x:

(42) $$H_{j}=\frac{\partial f(x)}{\partial x}{|}_{x=x_{j}}\;\;\;\mathrm{with}\;\;\;x_{0}=x^{-}.$$

In addition, the weighting of the observations arises in the *Kalman Matrix* K as follows:

(43) $$K_{j}=\Sigma_{\mathrm{xx}}^{-}\cdot H_{j}^{T}\cdot {\left(\Sigma_{\mathrm{BB}}+H_{j}\cdot \Sigma_{\mathrm{xx}}^{-}\cdot H_{j}^{T}\right)}^{-1},$$

where $\Sigma_{\mathrm{BB}}$ represents the absolute variance covariance matrix of the measurements on the base of the square values of the magnetic observations:

(44) $$\Sigma_{\mathrm{BB}}=\left(\begin{array}{ccc} B_{x}^{2} & 0 & 0\\ 0 & B_{y}^{2} & 0\\ 0 & 0 & B_{z}^{2} \end{array}\right).$$

Finally, the new state estimate of the *j*th iteration follows by the local magnetic field $B^{local}$ as follows [28]:

(45) $$x_{j+1}=x^{-}+K_{j}\cdot \left(B^{local}-f\left(x_{j}\right)-H_{j}\cdot \left(x^{-}-x_{j}\right)\right).$$

Until convergence is reached, steps (42) up to (45) are executed iteratively, so that the state update $x^{+}=x_{j+1}$ and its variance covariance matrix $\Sigma_{\mathrm{xx}}^{+}$ can be determined as follows:

(46) $$\Sigma_{\mathrm{xx}}^{+}=\left(I^{u\times u}-K_{j}\cdot H_{j}\right)\cdot \Sigma_{\mathrm{xx}}^{-}.$$

**Figure 10:**
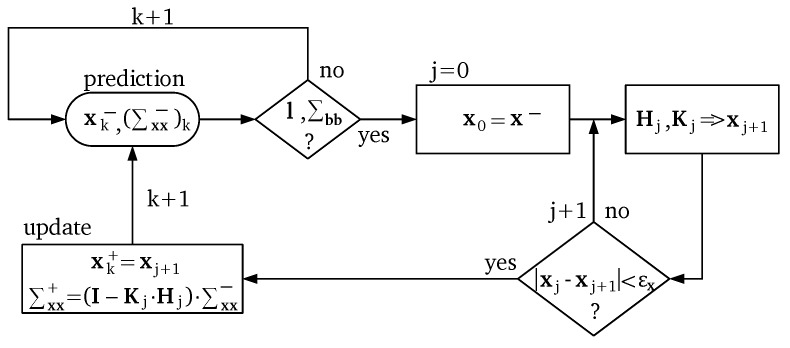
Flow diagram of the Iterated Kalman Filter (IKF).

Figure 10 illustrates the operating principle of the fusion algorithm in the form of a flow diagram.

## 5. Experimental Evaluation

Experimental measurements have been carried out using a mobile platform to test and evaluate the developed IMU/MILPS fusion algorithm for precise object tracking. This section describes the sensor platform and the experimental setup as well as the reached results.

### 5.1. Mobile Platform and Experimental Setup

**Figure 11:**
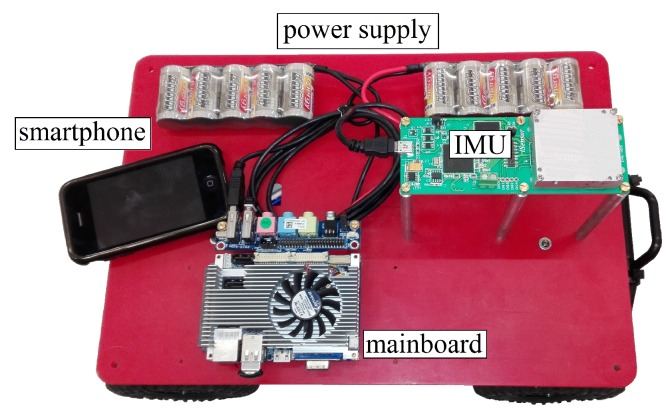
Remote-controlled platform.

The deployed vehicle, which is composed of a DC powered electric motor, can reach a maximum velocity of up to 3 m/s. It has a platform area of 0.3 × 0.4 m${}^{2}$ that enables mounting the Adis 16480 IMU as well as a mainboard for the data logging (see Figure 11). Information about the reached tracking accuracy is delivered by an iPhone 3 GS camera mounted at the object’s back site.

**Figure 12:**
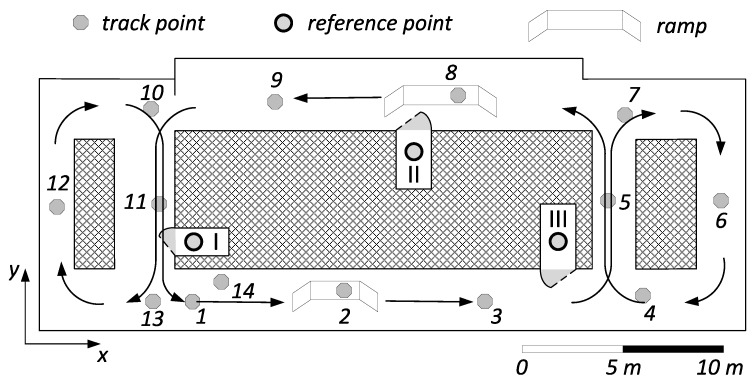
Positioning test area.

The tracking experiment was realized within a predefined indoor environment characterized by small corridors and separating concrete walls as part of the Technical University (TU) of Darmstadt institute building. The reference stations (coils) in the experiment illustrated in Figure 12 are placed in different rooms with thick walls, in order to show that MILPS in combination with IMU enables a continuous localization even in environments with obstacles and thick walls. This test area, which has a surface of about 440 m${}^{2}$, is composed of fourteen well-known tracking points that are marked with a reflecting foil at the ground floor as well as three magnetic coils. The tracking points and the reference stations realize the corresponding local coordinate frame, which is sketched in Figure 12. All points have been determined using a geodetic total station with a precision of less than 3 mm. Furthermore, two ramps are arranged to enable significant altitude changes.

The mobile platform is driven with a velocity of about 1 m/s along one closed loop. One round consists of the following consecutive track points:

1 - 2 - 3 - 5 - 7 - 6 - 4 - 5 - 8 - 9 - 11 - 13 - 12 - 10 - 11 - 1.

Additionally, an equivalent reference IMU is placed in a known height at the point 14 for observing the local ambient atmospheric pressure. All sensor data has been recorded in real time in the platform’s mainboard for a post-processing in MATLAB [29] to evaluate the implemented algorithm. The IMU sampling rate is set to 200 Hz and the activation time of the coils is 0.3 s. This ensures reaching high frequency inertial information as well as temporary magnetic field data, if the mobile platform is within the reach of the coils, which are placed at the height of one meter. The smartphone camera provides video sequences with a repetition rate of about 30 fps (frames per second), which enables a tracking acquisition of the passing points up to a resolution of 0.03 s. The common time stamp allows true-calculated comparisons of the corresponding track points at discrete locations. The IMU/smartphone synchronization is thereby carried out by matching significant movements in the video frames with the corresponding peaks of the acceleration signals. Furthermore, the camera recognizes the marked tracking points as true positions.

The post-processing applied to the stored sensor data includes the prediction step according to Section 4.3. A temporary update step is performed, if the MILPS-data are available (see Section 4.4). Therefore, the initial system state $x_{0}$, which consists of the starting point’s coordinates, the zero-velocity, the incipient orientation, as well as the state’s cofactor matrix $\Sigma_{0},$ must be predefined at k=0. The complete operation of the digital signal processing defined in Section 3.3.1 delivers inertial information as well as the corresponding variances. Additionally, height estimations are given by the measured air pressure evaluated by the adaptive filter method.

### 5.2. Experimental Results

**Figure 13:**
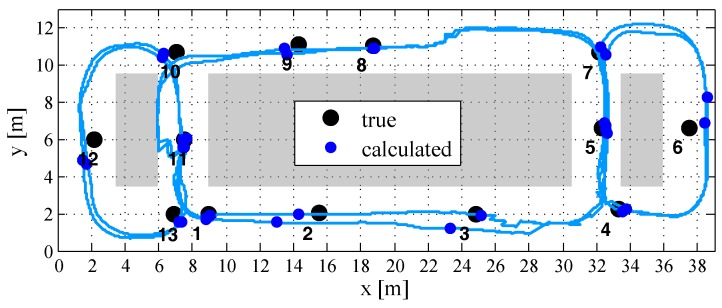
Tracking results of the x-y plane.

Although the coils are in the closed rooms I, II, and III with thick walls, the area outside these rooms is covered, which would not be the case by using UWB or ultrasonic signals (*cf.* Figure 12 and Figure 13). Figure 13 shows the tracking results of the whole test trajectory computed by using the IMU/MILPS sensor fusion in the *x*-*y* plane.

**Figure 14:**
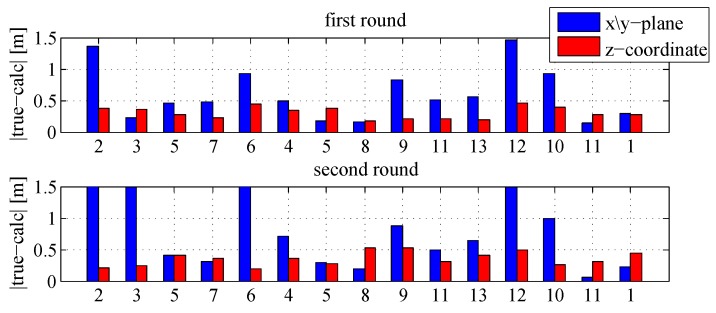
Comparison of the track points.

The calculated coordinates of the crossed track points, which are identified by the video captured from the smartphone camera, are illustrated with bold dots. The only use of the IMU solution is usually affected by significant drifts after a few seconds [30], in contrast to the utilized fusion algorithm enabling a stable and robust performance during the whole tracking phase with respect to an accuracy of less than one meter. The external magnetic field observations are not dependent from previous solutions, which makes them long-term stable and feasible to support data during the Kalman filter process. This statement is confirmed by the computed deviations between the true and the calculated track points in Figure 14.

Except for points 2, 3 and 6 within the second round, a maximum deviation of 1.5 m is not exceeded and the most parts of the measures are characterized by deviations of less than one meter. In addition, the relative air pressure observations yield to an altitude determination with an accuracy of less than 0.5 m. Hence, the applied barometer enables computations of the heights by the absence of the magnetic field signals.

**Figure 15:**
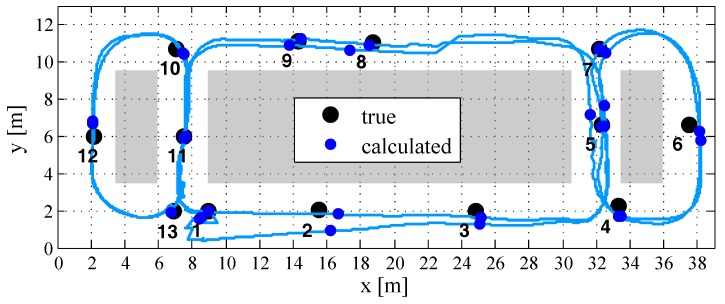
Tracking results (*x*-*y* plane) of the return path.

**Figure 16:**
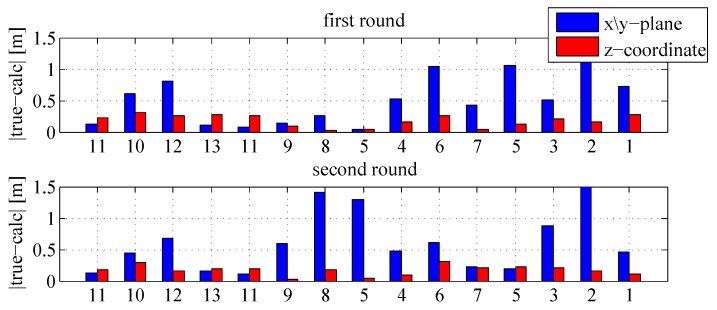
Comparison of the track points with the return path.

**Figure 17:**
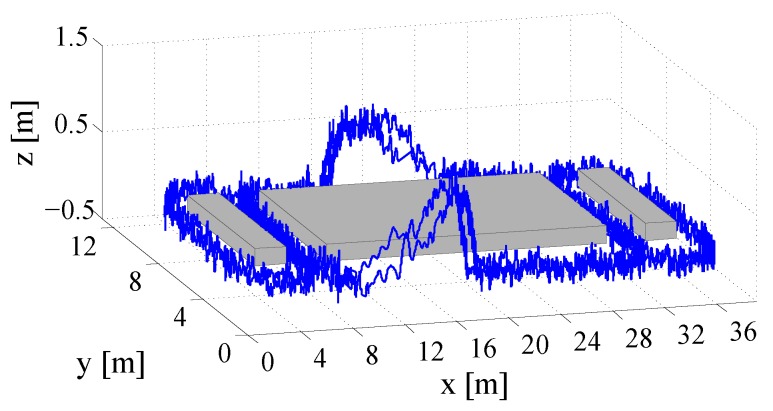
3D plot of the tracking paths.

To demonstrate the repeatability of the results, a second test drive has been carried out by passing through the return path, which is illustrated in Figure 15. This second measurement confirms the results of the first tracking test drive. The calculated trajectory differs from the true track in a range of about 1–1.5 m at the well-known track points (see Figure 16). The height-computations, which also confirm the results of the first test, have an accuracy of under 0.5 m. The results manifest that the developed tracking algorithm, which fuses inertial data with artificially generated magnetic fields, is feasible for localization in NLoS scenarios. The only use of inertial sensors suffers from position drifts in the range of a few meters after a short period. In contrast, the developed IMU/MILPS fusion enables solutions with accuracies of less than one meter in practical implementations. Figure 17 illustrates the three-dimensional presentation of the calculated tracking paths.

## 6. Conclusions

This contribution demonstrates the feasibility as well as the efficiency of the IMU/magnetometer fusion algorithm for indoor tracking of wheeled platforms. Therefore, the complete digital signal processing of the measured magnetic fields as well as the observed inertial data has been explained to realize the localization of industrial robots. The algorithmic part of this work includes the preprocessing of the measured time data, the motion modeling as well as the sensor data integration by applying the method of iterated Kalman filter. Furthermore, it is demonstrated that the assumption of suitable statistic parameters leads to good estimations of the system’s current state.

The experimental results, which are based on a realistic industrial platform within indoor-environments, demonstrate that the moving object can be localized with accuracies of less than 1.5 m in the horizontal plane and 0.5 m in *z*-direction. In addition, it is manifested that the altitude can be determined in an efficient manner by the deployment of pressure sensors in combination with the adaptive filtering technique. Finally, the reproducibility tracking tests show that the developed fusion algorithm enables a reliable as well as a precise object tracking in three-dimensional NLoS environments.

Further investigations should focus on more complex scenarios as well as usual velocity changes to show the algorithm’s robustness, in order to improve the system performance and stability.

## Figures and Tables

**Table 1:** Specification of ADIS 16480 [20].

	Dynamic Range	Resolution	Output Noise	In-Run Bias Stability	Random Walk
Accelerometer	±98.1 m/s${}^{2}$	1.2 × $10^{-8}$ m/s${}^{2}$	1.5 mg rms	0.1 mg	0.029 m/s/√hr
Gyroscope	±450${}^{\circ }$/s${}^{2}$	3.05 × $10^{-7}$${}^{\circ }$/s${}^{2}$	0.16 ${}^{\circ }$ rms	6.25${}^{\circ }$/hr	0.3 ${}^{\circ }\surd$hr
Magnetometer	±2.500 mGs	0.1 mGs	0.45 mgs	-	-
Barometer	(300–1100) mbar	6.1 × $10^{-7}$ mbar	0.025 mbar rms	-	-

## References

[1] Lecoq P. (2017). Pushing the limits in Time-Of-Flight PET imaging. IEEE Trans. Radiat. Plasma Med. Sci..

[2] Peng B., Kürner T. (2017). Three-Dimensional Angle of Arrival Estimation in Dynamic Indoor Terahertz Channels Using a Forward–Backward Algorithm. IEEE Trans. Veh. Technol..

[3] Meyer F., Tesei A., Win M.Z. Localization of multiple sources using time-difference of arrival measurements. Proceedings of the 2017 IEEE International Conference on Acoustics, Speech and Signal Processing (ICASSP).

[4] Mautz R. (2012). Indoor Positioning Technologies. Ph.D. Thesis.

[5] Norrdine A. (2009). Präzise Positionierung und Orientierung Innerhalb von Gebäuden. Ph.D. Thesis.

[6] Ubisense Ltd. The Ubisense Precise Real-time Location System. http://www.ubisense.net.

[7] Time Domain Corporation Precision Ranging & Location, Radar Sensing, Communications. http://www.timedomain.com.

[8] Hellmers H., Diefenbach N., Eichhorn A. (2016). IMU/UWB Sensorfusion fuer die Indoor-Positionierung von bewegten Plattformen. Zeitschrift fuer Vermessungswesen Heft.

[9] Want R., Hopper A., Falcão V., Gibbons J. (1992). The Active Badge Location System. ACM Trans. Inf. Syst..

[10] Gorostiza E.M., Galilea J.L.L., Meca F.J.M., Monzú D.S., Zapata F.E., Puerto L.P. (2011). Infrared sensor system for mobile-robot positioning in intelligent spaces. Sensors.

[11] Ziegler C. (1996). Entwicklung und Erprobung Eines Positionierungssystems für den Lokalen Anwendungsbereich.

[12] Medina C., Segura J.C., Holm S. Feasibility of ultrasound positioning based on signal strength. Proceedings of the 2012 International Conference on Indoor Positioning and Indoor Navigation (IPIN).

[13] Dabove P., Ghinamo G., Lingua A.M. (2015). Inertial sensors for smartphones navigation. SpringerPlus.

[14] Prigge E.A. (2004). A Positioning System with No Line-of-Sight Restrictions for Cluttered Environments. Ph.D.Thesis.

[15] Kasmi Z., Norrdine A., Blankenbach J. (2015). Towards a Decentralized Magnetic Indoor Positioning System. Sensors.

[16] Hellmers H., Norrdine A., Blankenbach J., Eichhorn A. An IMU/magnetometer-based Indoor positioning system using Kalman filtering. Proceedings of the International Conference on Indoor Positioning and Indoor Navigation.

[17] Hellmers H., Eichhorn A., Norrdine A., Blankenbach J. Indoor localisation for wheeled platforms based on IMU and artificially generated magnetic field. Proceedings of the 2014 Ubiquitous Positioning Indoor Navigation and Location Based Service (UPINLBS).

[18] Hellmers H., Eichhorn A., Norrdine A., Blankenbach J. IMU/magnetometer based 3D indoor positioning for wheeled platforms in NLoS scenarios. Proceedings of the 2016 International Conference on Indoor Positioning and Indoor Navigation (IPIN).

[19] Norrdine A., Kasmi Z., Blankenbach J. (2016). A novel method for overcoming the impact of spatially varying ambient magnetic fields on a DC magnetic field-based tracking system. J. Locat. Based Serv..

[20] Analog Devices, Inc. (2012). Ten Degree of Freedom Inertial Sensor with Dynamic Orientation Outputs–Datasheet ADIS 16480.

[21] Kreiss J.P. (2006). Einfuehrung in die Zeitreihenanalyse.

[22] Von Gruenigen D.C. (2008). Digitale Signalverarbeitung: Mit Einer Einfuehrung in Die Kontinuierlichen Signale und Systeme.

[23] Moschytz G.S., Hofbauer M. (2000). Adaptive Filter.

[24] Meschede D., Gerthsen C. (2010). Gerthsen Physik.

[25] Brannon R.M. (2002). ROTATION: A Review of Useful Theorems Involving Proper Orthogonal Matrices Referenced to Threedimensional Physical Space.

[26] Kuipers J.B. (2002). Quaternions and Rotation Sequences: A Primer with Applications to Orbits, Aerospace and Virtual Reality.

[27] Wendel J. (2011). Integrierte Navigationssysteme: Sensordatenfusion, GPS und Inertiale Navigation.

[28] Bell B.M., Cathey F.W. (1993). The iterated Kalman filter update as a Gauss-Newton method. IEEE Trans. Autom. Control.

[29] The Mathworks, Inc. (2015). MATLAB Version 8.5.0.197613 (R2015a).

[30] Klingbeil L., Romanovas M., Kolbe T.H., Bill R., Donaubauer A. (2014). Hybride Verfahren zur Indoor-Lokalisierung. Geoinformationssysteme 2014: Beitraege zur 1. Muenchner GI-Runde.

